# Empowering Healthcare Heroes: Unveiling the Impact of Self-Efficacy on Combating Outsider Mistreatment—A Systematic Review

**DOI:** 10.1155/jonm/7052173

**Published:** 2025-09-29

**Authors:** Elena Cavallari, Ilaria Setti, Valentina Sommovigo

**Affiliations:** ^1^Department of Brain and Behavioral Science, University of Pavia, Pavia 27100, Italy; ^2^Department of Psychology, Sapienza University of Rome, Rome 00185, Italy

**Keywords:** health personnel, incivility, occupational health, self-efficacy, systematic review, workplace violence

## Abstract

**Background:**

The healthcare sector is increasingly experiencing a rise in mistreatment from patients and visitors, a trend that has been intensified in recent years. This systematic literature review examines the relationship between outsider mistreatment and self-efficacy, a vital resource that helps professionals cope with socially stressful situations.

**Method:**

Following preferred reporting items for systematic reviews and meta-analysis guidelines, 24 quantitative observational and experimental studies, published between 2005 and 2024, were selected from the PsycInfo, PubMed, and Scopus databases through a structured search strategy. Eligibility for inclusion was established based on predefined criteria for both inclusion and exclusion. The methodological quality of the studies and the overall quality of evidence for the review were evaluated using the mixed methods appraisal tool and the grading of recommendations assessment, development, and evaluation, respectively. A narrative synthesis approach was employed to analyze and integrate the findings.

**Results:**

While the direct relationship between self-efficacy and mistreatment remains complex, self-efficacy—whether general or domain-specific—plays a protective role in mitigating the negative effects of outsider mistreatment on healthcare professionals' well-being in most studies. Interventions focusing on aggression management and enhancing self-efficacy have proven effective, with some studies reporting lasting benefits over time.

**Conclusion:**

This review represents the first comprehensive examination of the relationship between self-efficacy and outsider mistreatment in healthcare settings, emphasizing its role within a broader resource framework. A multifaceted approach, integrating self-efficacy-enhancing interventions with organizational strategies, is crucial for improving healthcare workers' well-being.

**Implications for Nursing Management:**

Experiential training in aggression management and emotional regulation can strengthen nurses' self-efficacy in handling aggressive patients or visitors. Beyond enforcing zero-tolerance policies and streamlined reporting systems, nursing leadership could provide support networks and debriefing opportunities. Combining personal and organizational strategies helps build a supportive work environment and a more resilient nursing workforce.

## 1. Introduction

In the healthcare sector, care and support are integral to daily interactions, making the relationship between health personnel and users (i.e., patients and their visitors) crucial. However, negative interactions have become increasingly common. Over the past 2 decades, healthcare workers have increasingly reported mistreatment from patients and visitors [1]. Estimates indicate that between 8% and 38% of healthcare workers encounter physical aggression during their careers, with many others subjected to verbal abuse [2]. Although these incidents are often underreported [3], they account for half of all workplace aggression [4], with patients and their families responsible for 93% of cases [5]. The COVID-19 pandemic has further intensified these issues, putting additional strain on healthcare systems and escalating user-perpetrated mistreatment worldwide [6]. Current trends suggest that conditions have yet to return to pre-pandemic levels [7].

Beyond overt aggression, subtler forms of mistreatment, such as incivility, are widespread [8]. Defined as “low-intensity deviant behavior with ambiguous intent to harm,” incivility encompasses gestures (e.g., snapping fingers for attention) and verbal slights (e.g., blaming staff for delays) [9]. While seemingly minor, repeated exposure can lead to cumulative psychological harm and may escalate into more severe mistreatment forms [8, 9]. To address the full spectrum of user-initiated misbehaviors—from incivility to verbal and physical aggression—the term “outsider mistreatment” is used, referring to any user-perpetrated conduct intended to harm healthcare workers [10].

### 1.1. Outsider Mistreatment and Self-Efficacy: An Integrated Perspective

The consequences of outsider mistreatment are significant, affecting individuals and organizations. For healthcare workers, such mistreatment poses a serious threat to their psycho-physical health, leading to issues, such as demoralization, anxiety, depression, burnout, and even posttraumatic stress symptoms [11, 12]. These adverse effects are closely tied to increased absenteeism, reduced job satisfaction, and impaired work performance, compromising the quality of patient care [13, 14]. On an organizational level, mistreatment results in workforce shortages and higher turnover rates, which hinder service delivery and access to care [15, 16].

When viewed through the lens of conservation of resources (COR) theory [17], outsider mistreatment emerges as a significant stressor that threatens or depletes vital resources. These resources include tangible objects (e.g., equipment), personal characteristics (e.g., self-worth), social conditions (e.g., feeling safe and respected at work), and energies (e.g., cognitive and emotional energies) [18]. COR theory posits that resource loss is more impactful than resource gain, triggering a downward spiral of cumulative losses. Without adequate recovery opportunities, this process can lead to increasing psychological strain and deteriorating occupational functioning [18]. According to COR theory [18], individual responses to outsider mistreatment vary based on the availability of personal resource, with self-efficacy being a central element. Self-efficacy is defined as the belief in one's ability to execute specific actions and cope with adversity [19], and it is a fundamental aspect of resilience and a key factor in how individuals interpret and manage stress [18]. Rooted in social cognitive theory (SCT, [20]), self-efficacy shapes perceptions of control over actions and environmental events, guiding behavior in professional settings. It empowers individuals to develop self-regulation strategies, enabling them to effectively manage stressful events [21]. Healthcare professionals with high self-efficacy are inclined to view outsider mistreatment as a manageable challenge rather than a threat [19, 21]. This adaptive appraisal mitigates physiological stress responses and reduces neuroendocrine reactivity to stressors, including mistreatment [22, 23]. Such individuals are also more likely to adopt proactive coping strategies (e.g., problem-solving techniques), which contribute to their sustained psychological well-being and job performance even in adverse conditions [18, 24]. Conversely, those with low self-efficacy are more vulnerable to the negative impacts of mistreatment, as they tend to perceive these experiences as uncontrollable threats [21]. This threat-based mindset heightens feelings of helplessness, escalates physiological arousal, and accelerates emotional exhaustion [25]. Their reduced ability to implement constructive coping strategies increases the risk of falling into a downward spiral of resource loss, which heightens their susceptibility to psychological malaise and diminished performance over time [26, 27].

Integrating COR and SCTs, self-efficacy is a critical personal resource that protects healthcare workers from the harmful effects of outsider mistreatment. High self-efficacy promotes resource replenishment, sustaining professional functioning even in the face of ongoing stressors. By shaping emotional responses and guiding adaptive coping behaviors, self-efficacy enhances individuals' abilities to effectively navigate challenging environments.

While the prevalence and effects of outsider mistreatment are well-documented [28, 29] and the importance of self-efficacy as a personal resource is widely recognized [21, 23, 27], there remains a significant gap in understanding how these constructs interact in healthcare settings. Existing reviews have examined outsider mistreatment and self-efficacy in isolation [1, 30, 31], but a systematic review focusing on their interrelationship has yet to be conducted. This oversight is particularly significant, as self-efficacy may protect against the stress induced by mistreatment. It is crucial to clarify how both general and domain-specific forms of self-efficacy impact stress responses and coping strategies in the face of outsider mistreatment. Such insights can inform targeted interventions aimed at enhancing healthcare workers' self-efficacy and supporting their well-being in challenging interpersonal environments. Therefore, this systematic review aims to synthesize and critically evaluate the existing evidence regarding the relationship between outsider mistreatment and self-efficacy among healthcare professionals.

### 1.2. Objectives

This systematic review aims to explore the relationship between self-efficacy and outsider mistreatment by synthesizing existing literature. It also evaluates the effectiveness of interventions designed to enhance healthcare workers' self-efficacy while addressing experiences of mistreatment. Specifically, the review focuses on the following questions:

1. How is healthcare workers' self-efficacy studied in relation to outsider mistreatment?
2. How do individual differences in self-efficacy shape healthcare workers' perceptions and responses to outsider mistreatment?
3. What interventions have been implemented to address outsider mistreatment among healthcare workers, while simultaneously considering or measuring self-efficacy?

By identifying potential protective effects and strategies for enhancing self-efficacy, this review aims to contribute to the well-being of healthcare workers and improve patient care outcomes.

## 2. Methods

The systematic review was conducted and reported between April and December 2024 by the preferred reporting items for systematic reviews and meta-analysis (PRISMA) guidelines [32]. The PRISMA checklist is provided in Supporting Table 1. Prior to conducting the review, a search of the international prospective register of systematic reviews (PROSPERO) database was performed, revealing no similar studies. Consequently, the review protocol was prospectively registered (CRD42024551809). In February 2025, the PROSPERO record was amended to update the review status, revise the title and objectives, and provide a more detailed specification of the inclusion and exclusion criteria. These modifications were intended to improve clarity and ensure alignment with the review's scope.

### 2.1. Eligibility Criteria

#### 2.1.1. Inclusion Criteria

To ensure consistency and clarity in the analysis, only quantitative studies were included in the review. These studies provide measurable and comparable data essential for assessing levels of self-efficacy and mistreatment, as well as evaluating their impact on healthcare workers. Eligible studies consisted of observational and experimental designs (cross-sectional or longitudinal) published in English in peer-reviewed journals. The study population comprised healthcare workers aged 18 and older who were employed in clinical settings. These environments offer structured organizational frameworks that facilitate systematic studies of mistreatment dynamics and interventions. The review included studies that explored the relationship between outsider mistreatment and self-efficacy (whether general or domain-specific), as well as assessing the relationship between self-efficacy and the psychological or organizational well-being of workers exposed to mistreatment. In addition, intervention studies aimed at reducing outsider mistreatment while considering the role of self-efficacy were also included.

#### 2.1.2. Exclusion Criteria

Studies conducted in informal settings (e.g., home healthcare) were excluded from this review. This exclusion was made to focus on outsider mistreatment occurring within clinical settings, where structured organizational environments with established protocols, policies, and support systems can significantly influence the experience and impact of such mistreatment. Furthermore, studies addressing sexual workplace violence, mobbing, bullying, or mistreatment from intra-organizational members (i.e., supervisors, co-workers) were omitted, as these forms of mistreatment involve different dynamics and require different interventions compared to outsider mistreatment. In addition, studies focused on the validation of scales or questionnaires, as well as secondary and tertiary literature, books, book chapters, abstracts, retracted articles, and gray literature, were also omitted from this review.

### 2.2. Identification

The database search included PubMed, PsychInfo, and Scopus. To capture a comprehensive range of relevant literature, variations in keywords and medical subject headings (MeSH) terms were utilized. These standardized terms, used by the National Library of Medicine, help in indexing and searching biomedical literature, ensuring the precise identification of relevant studies, even when different authors use varying terminology. The search strategy focused on three main conceptual areas: healthcare personnel, experiences of incivility and workplace aggression, and self-efficacy in its various forms. Alternative spellings and hyphenation variants were also considered. A detailed breakdown of the search strings used for each database is available in Supporting Table 2. This search was limited to studies published between 2005 and 2024, reflecting the introduction of workplace violence guidelines published by the International Labor Office (ILO), the World Health Organization (WHO), Public Services International (PSI), and the International Council of Nurses (ICN) [33].

### 2.3. Data Extraction

After selecting the records eligible for review, a characteristics table was designed to organize relevant information for each paper. This table includes the following details: (a) author(s) and publication year, (b) country of the study, (c) study design, (d) sample/setting, (e) observed variables, (f) measurement tools for outsider mistreatment and self-efficacy, (g) theoretical framework, and (h) main results.

### 2.4. Methodological Quality Assessment

The methodological quality of the included studies was evaluated using the mixed methods appraisal tool (MMAT), a validated instrument widely used in systematic reviews [34, 35]. MMAT was selected over other alternatives, such as the critical appraisal skills programme (CASP), because of its capacity to assess various quantitative study designs within a single framework, making it particularly well-suited for the objectives of this review. The assessment commenced with two screening questions focusing on the clarity of the research questions and the appropriateness of data collection methods. This was followed by five design-specific criteria that addressed sampling strategy, measurement validity, control for confounding variables, data completeness, and analysis appropriateness. Each item was rated as “Yes,” “No,” or “Can't tell,” in accordance with MMAT guidelines, which do not incorporate a numerical scoring system. The initial independent assessment was conducted independently by the first two authors, who documented the results along with comments. These results were then reviewed and discussed by the last author to resolve any discrepancies.

### 2.5. Data Synthesis and Certainty Assessment

A narrative synthesis approach was employed to integrate the findings, offering the flexibility needed to accommodate the heterogeneity of study designs, populations, and outcome measures. This approach also facilitated a nuanced interpretation of the evidence and enabled the consideration of practical implications for the healthcare sector. The synthesis focused on three main outcomes: (1) the relationship between healthcare workers' self-efficacy and experiences of outsider mistreatment; (2) the protective role of self-efficacy in promoting individual and occupational well-being; and (3) the effectiveness of interventions designed to mitigate mistreatment while concurrently enhancing self-efficacy. The certainty of evidence for each outcome was assessed using the grading of recommendations assessment, development and evaluation (GRADE) framework, which systematically assesses five domains: risk of bias, inconsistency, indirectness, imprecision, and publication bias.

## 3. Results

### 3.1. Screening

**Figure 1:**
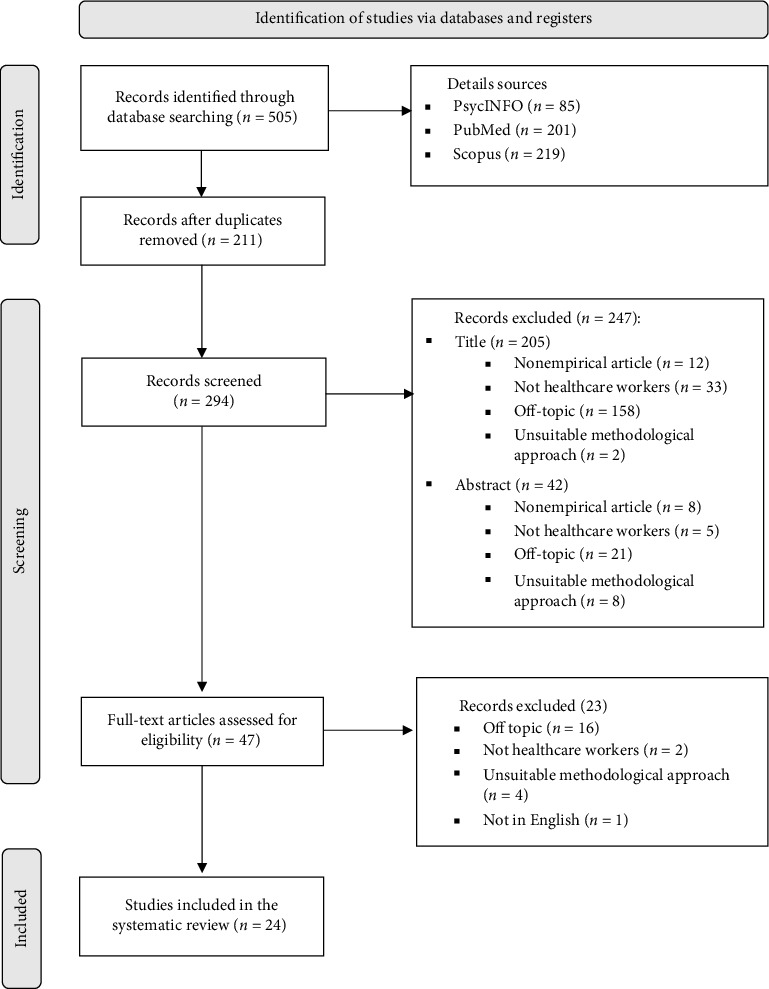
Flow diagram of records identified, screened, and included in the systematic review, according to Prisma Guidelines.

A total of 505 records were retrieved through database searches and imported into reference management software (Zotero and Mendeley).  Figure 1 illustrates the flowchart showing the number of papers identified, screened, and excluded at each stage of the process. Any uncertainties or disagreements about whether to include or exclude specific papers were resolved through discussions among the authors of the review. Details of studies excluded during the full-text screening stage, along with the reasons for their exclusion, are available in Supporting Table 3. Ultimately, 24 records met the criteria and were included in the review.

### 3.2. Study Characteristics

An overview of the study characteristics and key findings is provided in Table 1. The studies were conducted across various settings: 17 in primary and acute care facilities [36, 37, 40, 46, 51, 54, 58, 60, 61, 66, 67, 69, 71, 73, 75, 78, 83], 5 in residential care facilities and nursing homes [43, 45, 50, 63, 65], and 2 involved healthcare workers operating across different healthcare settings [62, 81]. Within primary and acute care facilities, five studies were conducted in general hospitals [40, 46, 61, 69, 78], four in emergency departments [67, 71, 73, 75], three in mental health wards [36, 37, 83], one in a neuroscience ward [66], one in a tertiary care children's hospital [60], and three involved both general and mental health settings [51, 54, 58]. Regarding job roles, 5 studies involved physicians [36, 40, 67, 75, 78], 5 focused on nurses [46, 61, 66, 69, 71], and 3 on nurse assistants [45, 63, 65], while 11 studies included a mixed sample of healthcare workers [48, 49, 52, 54, 60, 65, 72, 73, 85, 86].

Studies employed different theoretical frameworks: SCT [19] (*n* = 3 [45, 63, 65]), COR Theory [17] (*n* = 2; [58, 69]), job-demand resources (JD-R) model [49] (*n* = 2 [46, 81]), experiential learning theory (ELT) [74] (*n* = 2 [73, 83]), and theory of reasoned action (TRA) [64] (*n* = 2 [63, 65]). Other theories that were adopted included the demand-control-person (DCP) model [57] (*n* = 1 [54]), the failure mode and effects analysis (FMEA) model for improvement [72] (*n* = 1; [71]) and knowledge to action theory (KTA) [39] (*n* = 1; [37]). Eleven studies did not use any theoretical framework [36, 40, 43, 50, 51, 60–62, 66, 67, 75].

#### 3.2.1. Observational Studies

Most of the observational studies adopted a cross-sectional design (*n* = 11) [40, 43, 46, 51, 54, 58, 67, 69, 75, 78]. One study employed a closed cohort prospective design, which involved following a fixed group of participants over time to measure outcomes prospectively [62]. In addition, another study used a cross-lagged design that collected different variables at three distinct time points, with a 1 week interval between each [81].

Outsider mistreatment was mainly assessed as aggression across physical, verbal, and nonphysical dimensions (*n* = 10) [43, 46, 51, 54, 58, 61, 62, 67, 75, 78]; one study examined it as incivility [69]; one as verbal aggression and incivility [81]; one focused on patient contempt (i.e., disrespect and lack of cooperation from patients and families [40]).

Eight studies used validated self-reported scales to assess mistreatment [40, 46, 58, 69, 75, 78], while four employed ad hoc measures [61, 62, 67, 81]. One study assessed mistreatment based on aggression incidents reported by a staff manager [43]. Among the validated scales, three studies [51, 54, 58] used the mistreatment behaviors scale [52], consisting of six items on a six-point Likert scale; two studies [54, 58] utilized the mistreatment by patients and their families scale [55], comprising four items on a four-point Likert scale; one [69] study applied the workplace incivility scale (WIS) [70], with seven items on a five-point Likert scale; one [75] adopted the Chinese version of the effort–reward imbalance (ERI) [76], consisting of 23 items on a five-point Likert scale; one [78] used the workplace violence scale [79], consisting of 10 items on a four-point Likert scale; one [46] adopted the customer-related social stressor inventory [47], comprising 21 items on a four-point Likert scale; and one used the patient's contempt questionnaire [41], consisting of 13 items on a five-point Likert scale.

Self-efficacy was analyzed in its general form in five studies [62, 67, 75, 78, 81], and in two studies [40, 69] it was evaluated as a subdimension of the psychological capital, a pool of individual resources [42]. The other studies focused on specific domains of self-efficacy: managing challenging behaviors exhibited by patients (*n* = 2 [43, 61]), communication (*n* = 1 [46]), occupational coping (*n* = 1 [51]), emotion work (*n* = 1 [58]), and both occupational coping and emotion work (*n* = 1 [54]). Self-efficacy was evaluated using validated self-report scales in all studies except one, where the authors developed an ad hoc measure to evaluate levels of general self-efficacy [62]. Two studies [40, 69] used the psychological capital questionnaire (PCQ-24) [42], which comprises 24 items on a five-point Likert scale; four studies [67, 75, 78, 81] adopted the general self-efficacy scale, in its English [81] or Chinese translation [67, 75, 78], comprising 10 items on a four-point Likert scale; two studies [43, 61] employed the difficult behavior self-efficacy scale [44], consisting of five items on a seven-point Likert scale; two studies [51, 54] used the occupational coping self-efficacy questionnaire for nurses (OCSE-N) [53], consisting of nine items on a five-point Likert scale; and two studies [54, 58] utilized the emotion-work-related self-efficacy scale [56], comprising four items on a five-point Likert scale. Individual and occupational well-being was analyzed through both positive and negative outcomes. Positive outcomes included professional identity (*n* = 1 [40]), organizational citizenship behavior (*n* = 1 [69]), job satisfaction and job initiative (*n* = 1 [78]), and vitality and mental health (*n* = 1 [62]). Conversely, negative outcomes included burnout (i.e., emotional exhaustion, depersonalization, and reduced personal accomplishments (*n* = 4) [46, 51, 54, 58]), perceived medical errors (*n* = 1 [75]), occupational stress (*n* = 1 [78]), affective ill-being (*n* = 1 [81]), and somatic stress (*n* = 1 [62]).

A total of 11 studies investigated the relationship between mistreatment and self-efficacy, with most studies (*n* = 10 [40, 43, 46, 51, 54, 58, 61, 67, 78, 81]) focusing on correlations without establishing a clear directional link. Only one study tested self-efficacy as an antecedent to mistreatment [67], while one examined it as a covariate in a longitudinal analysis of mistreatment effects [62].

Ten studies analyzed the relationship between victimized healthcare worker's self-efficacy and well-being outcomes [40, 46, 51, 54, 58, 62, 69, 75, 78, 81], mainly exploring self-efficacy as a moderator in the relationship between mistreatment and these outcomes (*n* = 6 [46, 51, 54, 58, 78, 81]). In addition, three studies assessed self-efficacy as a predictor of well-being outcomes [40, 69, 75].

#### 3.2.2. Intervention Studies

Regarding the intervention studies included in the review (*n* = 11 [36, 37, 45, 50, 60, 63, 65, 66, 71, 73, 83]), three involved simulation-based learning [36, 73, 83], four focused on educational training [37, 45, 63, 65], one was a physical intervention program [66], and three were multifaceted interventions [50, 60, 71]. Five studies conducted assessments at three time points: pre-intervention, immediately post-intervention, and during follow-up [45, 50, 63, 73, 83]. In contrast, five studies assessed participants only before and after the intervention [36, 37, 60, 65, 66]. In one study, the effectiveness of the intervention was evaluated monthly over the subsequent 2 years [71]. Researchers predominantly employed self-report measures developed ad hoc for assessing the variable of interest. In all the studies, self-efficacy was evaluated in its specific components: managing aggression (*n* = 5 [36, 50, 63, 65, 71]), responding to violence (*n* = 1 [73]), knowledge and prevention skills (*n* = 1 [45]), confidence and safety around aggressive patients (*n* = 1 [66]), reporting and intervening (*n* = 1 [60]), management/leadership and communication/teamwork (*n* = 1 [83]), and in therapeutic communication (*n* = 1 [37]). Workplace mistreatment was assessed in four studies, primarily through the rate of assaults (*n* = 3 [37, 43, 45]), while one developed an ad hoc scale to assess an individual's response to events of violence [73].

### 3.3. Methodological Quality Assessment and Certainty Assessment

According to the MMAT guidelines, not all studies fully met the criteria for high or good quality (see Table 2). However, the decision was made to retain these studies in the analysis because of their relevance to the research questions. Moreover, the overall certainty of evidence across all key outcomes was rated as very low according to the GRADE framework (see Supporting Table 4). General self-efficacy was consistently associated with lower levels of outsider mistreatment, whereas domain-specific self-efficacy demonstrated more variable effects. These associations, however, were undermined by heterogeneity in measurement tools and contextual differences, leading to substantial imprecision. Higher self-efficacy was linked to improved occupational outcomes, but these findings were constrained by methodological limitations (e.g., reliance on self-reported outcomes, and the use of nonvalidated or ad hoc measures) and a lack of robust causal design. Outsider mistreatment management interventions, particularly those integrated with organizational support systems, showed promise in enhancing self-efficacy. Yet, the lack of validated outcome measures, small sample sizes, and limited follow-up periods reduce confidence in their sustained effectiveness.

## 4. Discussion

The analysis of the 24 studies included in this review provides critical insights into the multifaceted role of self-efficacy in protecting against outsider mistreatment in healthcare settings. Observational studies indicate that, while domain-specific self-efficacy has a limited direct impact on mistreatment, general self-efficacy is more consistently linked to fewer experiences of mistreatment—though the strength of its protective effects varies [40, 67, 78]. For instance, Meng et al. [67] observed that emergency physicians with high general self-efficacy experienced less verbal aggression, which was confirmed by the study conducted by Yao et al. [78].

Hogh et al. [62] further noted that prior exposure to aggression predicted future incidents, with general self-efficacy partially explaining this link. This broad form of self-efficacy may enhance resilience by helping healthcare workers view outsider mistreatment as more manageable [21, 27]. Conversely, domain-specific self-efficacy has shown weaker and less consistent associations with mistreatment [43, 46, 61]. For instance, Flynn et al. [43] demonstrated no statistically significant relationship between perceived self-efficacy and aggressive incidents in residential care environments. Hills [61] further corroborated these findings, noting that although aggression management training enhanced self-efficacy, it did not correspond with a reduction in aggression exposure over a longitudinal 3-year period. Consistent with these results, Goussinsky [51, 54] and Goussinsky and Livne [58] reported no meaningful association between occupational and emotion-work self-efficacy and the occurrence of mistreatment in general and psychiatric healthcare settings. Similarly, Gilardi et al. [46] observed no significant correlation between nurses' self-efficacy in patient communication and their experiences of verbal aggression.

These discrepancies may stem from methodological limitations, such as reliance on narrow assessment tools (e.g., the difficult behaviour self-efficacy scale [44]), which may not adequately capture the complexity of interpersonal dynamics involved in mistreatment. Furthermore, domain-specific self-efficacy may lack the broader cognitive and emotional scope needed to navigate the multifaceted and often unpredictable nature of outsider aggression in healthcare contexts [43, 46, 61]. Confidence in specific skills alone may not be sufficient to alter the frequency of adverse interactions with users. Instead, as research on workplace violence in healthcare settings has shown, the protective role of self-efficacy appears to be shaped by a constellation of factors, including staff attributes (e.g., younger age, limited work experience, and being a nurse) [28, 87], patient characteristics, environmental conditions (e.g., working in high-risk wards) [29], and the quality of staff–patient relationships [85].

In spite of variability in its direct impact on mistreatment, self-efficacy emerges as a critical resource for healthcare workers' well-being in most studies. In the context of outsider mistreatment, higher self-efficacy is associated with lower levels of occupational stress and burnout [46, 51, 54, 58], as well as positive job outcomes, including increased job satisfaction, fewer medical errors, and enhanced organizational citizenship behavior [69, 75, 78]. For instance, Yao et al. [78] found that general self-efficacy helped buffer the effects of experienced and witnessed aggression on occupational stress and job satisfaction. Drawing on COR [18] and SCTs [21], self-efficacy—encompassing self-confidence, perceived competence, and an internal locus of control—plays a key role in facilitating adaptive coping and self-regulation. Healthcare workers with high self-efficacy tend to be more emotionally stable, confident in managing conflicts, and less likely to escalate situations during incidents of mistreatment [67]. In contrast, those with lower self-efficacy are more susceptible to outsider mistreatment and its negative psychological effects [24, 88], including occupational stress [78] and burnout symptoms [46, 51, 54, 58]. Overall, all included studies consistently highlighted the detrimental impact of outsider mistreatment on healthcare worker's psycho-physical well-being and work functioning, in line with previous literature [86]. Moreover, the findings align with a substantial body of research indicating that individuals with high self-efficacy tend to perceive themselves as capable of effectively managing challenging situations [27, 31, 89]. This belief enhances their sense of control, fosters a challenge-oriented appraisal of stressors as manageable, and supports adaptive functioning, contributing to more favorable well-being outcomes [27, 89]. However, the protective role of self-efficacy is neither consistent nor guaranteed. For instance, Deng et al. [40] found that general practitioners who faced patient contempt reported a diminished sense of professional identity, even when self-efficacy was high. In some instances, elevated self-efficacy may paradoxically contribute to maladaptive outcomes, such as feelings of helplessness, particularly when healthcare workers face unpredictable stressors [81]. Healthcare workers with high self-efficacy may interpret mistreatment as a reflection of their own inadequacy, which can undermine its otherwise protective function [85]. These findings challenge the long-standing “more is better” perspective on self-efficacy by contributing to a growing body of evidence that “too much” self-efficacy may yield detrimental effects [90].

Specifically, overly elevated self-efficacy can foster an inflated sense of competence and diminish the perceived need for sustained effort, leading to complacency, reduced effort, and compromised work outcomes [90]. In addition, prolonged exposure to mistreatment can erode self-efficacy's benefits; for instance, Yao et al. [78] found that its benefits diminished with increasing aggression, while Yaranon et al. [81] reported that it did not significantly moderate the relationship between mistreatment and affective ill-being. These findings suggest that, rather than acting as a consistent protective factor, self-efficacy may serve as a vulnerability factor when low or may not serve as a universal remedy for all stressors—particularly when its domain-specific aspects are not aligned with the critical tasks necessary to manage mistreatment effectively.

Crucially, self-efficacy does not function in isolation; its protective effects are significantly enhanced when complemented by additional job resources. For instance, Gilardi et al. [46] found that while self-efficacy positively correlated with role clarity, only role clarity moderated the effects of verbal aggression on burnout. Similarly, Goussinsky and Livne [58] reported that job autonomy—rather than self-efficacy alone—buffered the impact of mistreatment on burnout. Healthcare workers with low self-efficacy and limited access to contextual resources, such as co-worker support or job autonomy, were more susceptible to emotional exhaustion and depersonalization caused by mistreatment. In contrast, those with high self-efficacy and sufficient resources reported more favorable psychological outcomes [51, 54]. These findings align with COR theory, particularly the notions of “resource caravans” (i.e., resources accumulate and reinforce one another over time) and “resource caravan passageways” (i.e., workplace conditions that support, enrich, and safeguard individuals' resources; [18, 91]). Together, these mechanisms support gain spirals (i.e., self-sustaining cycles of resource enrichment; [18]). Thus, the protective benefits of self-efficacy appear to be contingent on the presence of the complementary job resources. Rather than functioning in isolation, self-efficacy exerts its positive effects synergically with organizational resources (e.g., social support) that amplify its capacity to buffer against stressors and promote adaptive outcomes.

Intervention studies highlight the potential of targeted training programs to enhance healthcare workers' self-efficacy in managing outsider mistreatment. Simulation-based training, especially in high-risk contexts, has demonstrated substantial benefits by integrating technical and nontechnical skills—such as communication and emotion regulation—thereby enhancing self-efficacy and aggression management competencies [36, 50, 73, 83]. For instance, Ajaz et al. [36] reported increased confidence among psychiatric and general healthcare trainees. Similarly, Young et al. [83] found sustained improvements in leadership, communication, and teamwork among mental health professionals 3 months post-training. In addition, emergency violence simulations combined with preventive workshops significantly improved healthcare workers' self-efficacy and responsiveness to mistreatment, with these gains maintained at a 2-week follow-up [73]. These findings align with those of a previous systematic review, which reported that acute healthcare staff who participated in simulation-based training for managing clinical aggression demonstrated significant gains in knowledge and self-reported confidence [92]. Moreover, educational interventions incorporating de-escalation strategies and physical training programs have been found to improve healthcare workers' confidence, problem-solving abilities, and responses to mistreatment [37, 45, 50, 63, 65, 66]. For instance, Irvine et al. [63, 65] developed multimedia-based programs based on de-escalation techniques that enhanced nurse assistants' self-efficacy, empathy, and behavioral intentions toward aggressive residents with effects sustained at a 2-month follow-up [63]. Likewise, therapeutic communication training for mental health nurses not only boosted self-efficacy and knowledge but also contributed to a reduction in aggressive incidents [85]. Realistic simulations, with structured feedback and debriefing sessions, further reinforced participants' capacity to handle aggressive situations [36, 73, 83]. Programs emphasizing interpersonal skills, de-escalation strategies, and problem solving consistently yielded favorable outcomes [63, 65, 85]. Moreover, physical training, particularly in breakaway techniques, enhanced self-efficacy and safety in high-risk environments [66]. For example, Lamont et al. [66] found that these programs increased the confidence and sense of safety among nurses, with the positive effects lasting up to 8 weeks after the intervention.

While self-efficacy-enhancing interventions have demonstrated initial effectiveness, their long-term sustainability remains a challenge, as gains in self-efficacy often diminish over time without continued reinforcement [45, 50]. For instance, violence-prevention programs have shown short-term benefits but limited long-term impact. Gates et al. [45] reported initial gains in self-efficacy and knowledge, with reductions in assaults confined to those that were infrequent before the intervention; however, these effects significantly declined by 6 months. Similarly, Gordon et al. [50] found that although educational and simulation-based training temporarily improved confidence and triggered recognition, these improvements faded over time, yielding no lasting decrease in aggressive incidents. Consistent with previous studies [85], multifaceted approaches that integrate individual-level training with organizational strategies have shown greater long-term efficacy. For instance, Hatfield et al. [60] implemented a program in a children's hospital that integrated inter-professional training with revised reporting and response protocols, leading to increased self-efficacy, improved policy awareness, and a threefold rise in incident reporting. Similarly, Okundolor et al. [71] applied a comprehensive approach—including inter-professional education, crisis drills, and support systems—which significantly enhanced self-efficacy and contributed to a marked reduction in harmful assaults within emergency psychiatric settings. These findings underscore the value of evidence-based interventions in enhancing healthcare workers' self-efficacy and decreasing instances of mistreatment. However, their long-term effectiveness remains uncertain, as some studies have indicated a decline in impact over time [45, 50].

Addressing mistreatment in healthcare requires a multifaceted approach, as self-efficacy alone is insufficient to counteract its detrimental effects. While self-efficacy serves as a critical personal resource that helps prevent resource depletion because of outsider mistreatment [91], its protective capacity is significantly amplified in resource-rich environments—those characterized by supportive workplace conditions [91]. In contrast, within resource-poor settings or under conditions of intense mistreatment, self-efficacy alone may fail to prevent downward spirals of resource loss, highlighting the imperative for systemic interventions that bolster individual and contextual resources. Drawing on COR theory, effective interventions should integrate self-efficacy-enhancing training with organizational initiatives aimed at cultivating environments that support resource accumulation. At the individual level, training programs that encourage adaptive behaviors and correct maladaptive responses have demonstrated effectiveness in strengthening self-efficacy and promoting sustained behavioral change [36, 45, 63, 65, 66, 73, 83]. However, without supportive workplace structures, these individual-level gains may diminish over time. At the organizational level, interventions such as structured debriefings, peer support groups, and updated reporting systems contribute to a culture that proactively addresses mistreatment and reinforces healthcare workers' perceived ability to manage challenging interactions [60, 71]. Thus, self-efficacy should not be seen as a standalone solution but rather as a vital component of a broader intervention strategy that combines individual development with resource-rich work environments. Self-efficacy should be understood not as a standalone remedy but as a crucial element within a broader, integrated strategy. A multifaceted approach that integrates self-efficacy enhancement with robust workplace support systems and organizational practices is essential for fostering a resilient workforce in healthcare settings.

The methodological assessment revealed significant limitations in the included studies, which greatly impacted the overall quality and certainty of the evidence. Although all studies were kept for their conceptual relevance to the research questions, many did not fully meet the quality criteria established by the MMAT. Common methodological issues included the use of nonvalidated measures, reliance on self-reported outcomes that were susceptible to social desirability and recall bias, small sample sizes, and a lack of long-term follow-up assessments. In addition, the absence of control groups and the limited use of longitudinal designs significantly hindered the ability to establish causal relationships. These methodological challenges were reflected in the GRADE certainty assessment, which rated the evidence for all key outcomes as very low. Specifically, significant imprecision because of variability in measurement tools and contextual differences across healthcare settings diminished confidence in the observed effects. Nonetheless, the inclusion of these studies was justified based on their conceptual relevance and the emerging nature of the topic. GRADE assesses the quality—not the relevance—of evidence, and excluding methodologically limited studies in underdeveloped research areas risks omitting valuable contextual insights. Retaining all eligible studies enabled a comprehensive and transparent synthesis, while highlighting the need for future research to adopt more rigorous, theory-driven designs, standardized measures, longitudinal designs capable of establishing causal links, and robust sampling to strengthen the evidence base and inform effective interventions in healthcare settings.

## 5. Strengths and Limitations

This systematic review represents the first comprehensive examination of the role of self-efficacy in relation to outsider mistreatment within healthcare settings. By incorporating studies from various healthcare contexts, professional roles, and cultural backgrounds, it offers a broad and integrative perspective. Methodologically, the review is bolstered by strict adherence to PRISMA guidelines and the inclusion of diverse study designs, including intervention-based research. Furthermore, the use of validated tools such as the MMAT and the GRADE approach enhances the transparency, quality appraisal, and overall reliability of synthesized evidence.

In spite of its contributions, this review has several limitations that should be acknowledged to contextualize the findings. First, while it addresses incivility and overt aggression, these forms of mistreatment—characterized by varying intensity, persistence, and underlying motivations [10]—may have distinct relationships with self-efficacy that are not fully disentangled in the current synthesis. Second, by focusing exclusively on mistreatment from external sources, the review overlooks internally driven mistreatment such as bullying, harassment, and abusive supervision. From a methodological standpoint, many included studies relied heavily on self-reported data, which are vulnerable to biases such as social desirability and recall inaccuracies. Furthermore, several intervention studies used ad hoc or nonvalidated measures, raising concerns regarding the reliability and construct validity of the reported outcomes. Experimental designs frequently exhibited methodological shortcomings, including small sample sizes, absence of control groups, and lack of longitudinal follow-up, all of which constrain the robustness and generalizability of the findings. These limitations are reflected in the GRADE assessment, which rated the certainty of evidence as very low across all key outcomes. Collectively, these factors substantially diminish confidence in the synthesized results and highlight the urgent need for more methodologically rigorous and standardized research in this domain. Furthermore, existing interventions have largely concentrated on managing aggression from patients, with minimal attention paid to mistreatment by visitors—an equally critical yet underexplored challenge for healthcare professionals [5]. Another limitation lies in the difficulty of isolating the unique role of self-efficacy in mitigating the impact of outsider mistreatment. Given that many studies examined self-efficacy in conjunction with other psychological or contextual variables, determining its specific contribution remains problematic. The review also acknowledges a potential language bias because of the restriction to English-language publications, which may have excluded relevant research in other languages. Most studies reviewed were published after 2005, aligning with the introduction of major international workplace violence prevention frameworks. While this focus enhances contemporary relevance, it may overlook earlier work that could shed light on the link between self-efficacy and mistreatment. Finally, the diversity of theoretical frameworks employed across studies has limited the comparability of findings.

## 6. Implications for Researchers

This review emphasizes the distinct roles of general and domain-specific self-efficacy in shielding healthcare workers from outsider mistreatment. General self-efficacy appears to be a more reliable protective factor, while domain-specific self-efficacy has shown weaker and less consistent associations. These findings highlight the need to move beyond a one-size-fits-all understanding of self-efficacy and to examine its contextual relevance more thoroughly. Importantly, self-efficacy should not be regarded as a universal defense against mistreatment. While low self-efficacy can increase vulnerability, high self-efficacy—especially when narrowly defined—may provide little protection unless it aligns with the specific interpersonal and emotional challenges of the healthcare environment. This suggests a shift in focus, from isolated psychological traits to an integrated framework where self-efficacy works alongside complementary individual and organizational resources. Future research should explore how self-efficacy interacts with other personal and organizational resources, and how these combinations impact healthcare workers' capacity to endure mistreatment. Future research should also investigate the combined impact of insider and outsider mistreatment on healthcare workers' self-efficacy and well-being. These insider dynamics—such as bullying or abusive supervision—may interact with or exacerbate the effects of outsider mistreatment, compounding psychological strain and undermining personal resources. Multisource or multilevel designs could help clarify these interaction effects and illuminate whether certain personal and organizational resources buffer against this cumulative burden. Furthermore, the ongoing underrepresentation of incivility in the mistreatment literature—in spite of its high prevalence—reveals a significant blind spot. Differentiating between various forms of mistreatment and aligning them with the most relevant types of self-efficacy could enhance conceptual clarity. To support this effort, future research would benefit from being grounded in unified theoretical models, while also developing new, context-specific frameworks that consider variability across healthcare settings.

Methodologically, advancing this field requires stronger research designs, such as longitudinal and experience-sampling methodologies. Evidence indicating that daily mistreatment negatively affects self-perceptions (e.g., self-esteem [93]) warrants further investigation into whether positive day-to-day experiences can help restore self-efficacy in the short term. Examining fluctuations in daily self-efficacy may uncover new opportunities for timely and targeted interventions. Randomized controlled trials and quasi-experimental studies with adequate sample sizes and control conditions are crucial for testing the effectiveness of interventions. Importantly, mistreatment from visitors should also be accounted for in future intervention designs. Researchers should prioritize the use of psychometrically validated instruments and multisource assessments (e.g., supervisor ratings) to reduce bias and enhance reliability. Lastly, future reviews should broaden their scope by including studies published in multiple languages. This would incorporate evidence from non-English-speaking contexts and enrich the global understanding of these dynamics. Comparative research across different occupational roles and clinical environments is also necessary to determine whether certain forms of domain-specific self-efficacy are more protective in some settings than others.

## 7. Implications for Nursing Education and Management

The findings of this review have significant implications for nursing education, clinical practice, and nursing management. While higher self-efficacy is generally associated with greater resilience in the face of outsider mistreatment, mixed findings suggest that self-efficacy alone is not a universal safeguard. Its effectiveness is maximized when reinforced by a broader network of organizational resources. Consistent with the COR theory [18], this review advocates for multilevel, systemic approaches to improve individual and contextual resilience among nurses.

### 7.1. Simulation-Based and Experiential Learning

Nursing education should incorporate simulation-based learning strategies, such as role-playing, scenario-based training, and virtual simulations, to enhance domain-specific self-efficacy by providing experiential mastery in managing aggressive interactions [36, 73, 83].

### 7.2. Communication and Conflict Resolution Training

The curriculum should explicitly address key interpersonal skills, such as de-escalation, assertive communication, conflict resolution, emotional regulation, and breakaway training, to build professional self-efficacy in high-stakes clinical encounters [37, 63, 65, 66]. Moreover, structured coaching, feedback, and debriefing sessions can further strengthen self-efficacy [36, 37, 60, 71, 83].

### 7.3. Ongoing Professional Development

Given the dynamic nature of self-efficacy healthcare environments, training on self-efficacy and mistreatment management should extend beyond pre-licensure education. Ongoing learning through workshops, online modules, and interprofessional training can help maintain and enhance nurses' self-efficacy over time.

### 7.4. Multilevel Interventions for Resource Cultivation

In line with the COR theory, nursing management should implement a dual strategy that fosters both personal and environmental resources. Creating “resource caravans” and “resource caravan passageways” ensures that nurses receive ongoing support in the face of outsider mistreatment.

### 7.5. Supportive Leadership and Peer Networks

Supervisors and managers should be trained to support staff during and after incidents of outsider mistreatment [83]. Systems for peer support, psychological safety measures, and debriefing opportunities should be systematically integrated into clinical units [60, 71].

### 7.6. Policy Enforcement and Reporting Mechanisms

Implementing transparent, enforceable zero-tolerance policies against outsider mistreatment, alongside streamlined reporting procedures, can cultivate a safe workplace [60, 71]. Such mechanisms protect nurses and reinforce their belief in the organization's commitment to their well-being.

### 7.7. Tailored Interventions Based on Context and Role

Because of differences in mistreatment exposure across wards and professional roles, interventions should be targeted to specific occupational groups. Nursing managers could examine how domain-specific self-efficacy relates to mistreatment patterns in diverse settings to design support systems.

### 7.8. Promoting a Resilient and Inclusive Work Culture

A holistic approach integrating individual and organizational interventions can create resource-rich work ecosystems that protect nurses from outsider mistreatment while strengthening their self-efficacy. Prioritizing emotional and structural support enables nursing management to mitigate the impact of outsider mistreatment and support high-quality care under pressure.

## 8. Conclusion

While general self-efficacy has been associated with reduced exposure to mistreatment, evidence remains inconsistent, particularly regarding domain-specific self-efficacy. Crucially, self-efficacy does not operate in isolation but is most effective when reinforced by workplace resources.

To foster healthcare worker resilience, self-efficacy must be integrated into a broader, multilevel approach that combines targeted individual training with systemic organizational strategies. By embedding self-efficacy within resource-rich environments, healthcare organizations can enhance resilience, improve well-being, and cultivate a healthier workforce.

## Figures and Tables

**Table 1:** Summary of findings on the relationship between self-efficacy, outsider mistreatment, and individual or occupational constructs.

Author (s), year	Country	Study design	Subjects/setting (n)	Observed variables		Measures		Theoretical framework	Main results
				Variables of interest	Additional variables	Workplace mistreatment	Self-efficacy		
1. Ajaz et al., 2016 [36]	England	Nonrandomized controlled trials	General health and psychiatric trainees (9)	Simulation training Confidence in managing aggression	—	Items developed by the authors	Items developed by the authors	No theoretical framework was adopted	The intervention improved confidence in managing aggression, as assessed immediately after the training.

2. Amara et al., 2024 [37]	USA	Nonrandomized controlled trials	Nurses and health technicians in psychiatry (15)	Education and training in therapeutic communication Violent incidents Self-efficacy in therapeutic communication	Knowledge in therapeutic communication	Incident report	Self-efficacy in clinical communication skills (SE) [38]	Knowledge-to-action (KTA) [39]	The intervention decreased violence incidents and increased self-efficacy levels.

3. Deng et al., 2023 [40]	China	Cross-sectional	General practitioners in a primary healthcare center (2180)	Patient contempt Self-efficacy Professional identity	Psychological capital (resilience, optimism, hope)	Patient contempt questionnaire [41]	Psychological Capital Questionnaire [42]	No theoretical framework was adopted	Patient contempt was negatively associated with professional identity. Self-efficacy was positively associated with patient contempt and negatively associated with professional identity.

4. Flynn et al., 2018 [43]	United Kingdom	Cross-sectional	Residential staff for people with intellectual disabilities (186)	Aggressive challenging behavior Self-efficacy in managing challenging behavior	Burnout symptoms, Empathy, Overall positive contributions	Incidents reported by the staff manager	Difficult behavior self-efficacy scale [44]	No theoretical framework was adopted	No association was found between exposure to aggressive behaviors and self-efficacy, nor between exposure to aggression and the other variables. Clustering effects between residential facilities were observed for emotional exhaustion and work motivation.

5. Gates et al., 2005 [45]	USA	Nonrandomized controlled trials	Nurse assistants in a nursing home (138)	Educational training Incidence of assaults Self-efficacy in preventing aggression	Knowledge to prevent aggression Violence-prevention skills State and trait anger	Incidence of assaults	Items developed by the authors	Social cognitive theory (SCT) [19]	One week after the intervention, knowledge, self-efficacy, and skills in managing aggression levels increased. However, no significant difference in self-efficacy and prevention levels was observed 6 months after the intervention. The intervention significantly decreased the incidence of assaults among those who had experienced fewer than five assaults before the intervention.

6. Gilardi et al., 2020 [46]	Italy	Cross-sectional	Nurses in a general hospital (356)	Verbal aggression Self-efficacy in communication with patients Emotion work Burnout symptoms	Role clarity	Customer-related social stressor inventory [47]	The nurse's communication perceived self-efficacy scale [48]	Job-demand resources (JD-R) model [49]	Self-efficacy was negatively associated with emotional exhaustion and positively related to role clarity. The indirect effect of verbal aggression on burnout through emotion work was moderated by role clarity.

7. Gordon et al., 2021 [50]	Australia	Nonrandomized controlled trials	Residential and community aged care workers (17)	Educational and simulation training Incidence of aggression Self-efficacy in managing aggression	—	Items developed by the authors	Items developed by the authors	No theoretical framework was adopted	Self-efficacy levels significantly increased 1 week after the intervention, with this effect maintained at the 6-month follow-up. The intervention did not affect the overall incidence of aggression.

8. Goussinsky, 2020 [51]	Israel	Cross-sectional	Sample 1: Nurses in a general hospital (105) Sample 2: Healthcare workers in a mental health hospital (234)	Verbal mistreatment Occupational coping self-efficacy Burnout symptoms	Co-worker support	Mistreatment behaviors scale [52]	Occupational coping self-efficacy questionnaire for nurses [53]	No theoretical framework was adopted	Mistreatment was positively associated with emotional exhaustion and depersonalization in both samples. Self-efficacy was negatively associated with depersonalization only in sample 2 and positively associated with coworker support. The three-way interaction of self-efficacy, co-worker support, and mistreatment in predicting depersonalization was significant in both samples

9. Goussinsky, 2023 [54]	Israel	Cross-sectional	Sample 1: Nurses in a general hospital (217) Sample 2: Healthcare workers in a mental health hospital (309)	Nonphysical aggression Emotion-work self-efficacy (sample 1) Occupational coping self-efficacy (sample 2) Burnout symptoms	Job autonomy	Nonphysical aggression scales [52, 55]	Emotion-Work Related Self-Efficacy Scale [56] Occupational Coping Self-Efficacy Questionnaire for Nurses [53]	Demand-control-person (DCP) model [57]	Nonphysical aggression was positively associated with emotional exhaustion and depersonalization in both samples. Occupational coping self-efficacy was negatively associated with emotional exhaustion and depersonalization and positively related to job autonomy. The interaction of mistreatment, self-efficacy, and job autonomy on depersonalization was significant in both samples.

10. Goussinsky and Livne, 2019 [58]	Israel	Cross-sectional	Sample 1: Nurses in a general hospital (217) Sample 2: Healthcare workers in a mental health hospital (234)	Mistreatment Emotion-work Self-efficacy Burnout symptoms	Job autonomy	Mistreatment behaviors [55] Challenging client behavior and circumstances questionnaire [59]	Emotion-work-related self-efficacy scale [56]		Mistreatment was negatively associated with emotional exhaustion and depersonalization in both samples. Self-efficacy was negatively associated with emotional exhaustion and depersonalization only in sample 2 and positively associated with job autonomy. Job autonomy moderated the association between mistreatment and depersonalization.

11. Hatfield et al., 2022 [60]	USA	Nonrandomized controlled trials	Healthcare workers in a tertiary care children hospital (309)	Organizational intervention Mistreatment Self-efficacy in reporting and managing mistreatment	Knowledge of mistreatment policies	Items developed by the authors	Items developed by the authors	No theoretical framework was adopted	The intervention significantly increased healthcare professionals' self-efficacy in handling mistreatment and their knowledge of mistreatment policies and tripled the frequency of incident reporting

12. Hills, 2008 [61]	Australia	Cross-sectional	Nurses in a general rural hospital (300)	Aggression (in the past 3 months) Self-efficacy in managing aggression	Training in managing aggression (in the previous 5 years)	Items developed by the authors	Difficult behavior self-efficacy scale [44]	No theoretical framework was adopted	Training within the previous 5 years was weakly negatively associated with verbal abuse and weakly positively associated with self-efficacy.

13. Hogh et al., 2008 [62]	Denmark	Cohort study	Healthcare workers in primary and residential care facilities (2847)	Aggression General self-efficacy	Sense of coherence Vitality (energy, fatigue) Mental health (feelings of nervousness, depression, happiness and peacefulness) Somatic stress	Items developed by the authors	Items developed by the authors	No theoretical framework was adopted	Previous exposure to aggression significantly predicted exposure to aggression at follow-up (1 year later) and had a small but significant impact on somatic stress and mental health. Self-efficacy, together with other covariates, accounted for 10% of the association between previous exposure to violence and health outcomes at follow-up.

14. Irvine et al., 2012 [63]	USA	Randomized controlled trials	Nurse assistants in residential care (159)	Online educational training Self-efficacy in managing aggression	Self-efficacy in training scenarios Response knowledge in training scenarios Attitudes toward aggressive resident actions Empathy toward resident User acceptance	Items developed by the authors	Items developed by the authors	Social cognitive theory (SCT) [19] Theory of reasoned action (TRA) [64]	The training significantly increased levels of self-efficacy, both at T2 (2 weeks after the training) and at T3 (2 months later). Time invested in training was positively associated with levels of self-efficacy. Empathy and attitudes increased at T2, but empathy levels declined at T3.

15. Irvine et al., 2007 [65]	USA	Randomized controlled trials	Nurse assistants in residential care (62)	Online educational training Self-efficacy in managing aggression	Self-efficacy in training scenarios Response knowledge in training scenarios Attitudes toward aggressive actions Behavioral intention toward aggressive actions User acceptance	Items developed by the authors	Items developed by the authors	Social cognitive theory (SCT) [19] Theory of reasoned action (TRA) [64]	The training led to a significant increase in self-efficacy levels at T2 (one day after the training). Similarly, all other variables showed an increase.

16. Lamont et al., 2012 [66]	UK	Nonrandomized controlled trials	Nurses in neuroscience (32)	Breakaway training Confidence and safety around aggressive patients and in managing aggression	Exposure and confidence in breakaway domains (hand grabs, clothing grabs, hair pulls, chokes, strangles, and bear hugs)	Items developed by the authors	Items developed by the authors	No theoretical framework was adopted	The training significantly increased confidence and safety ratings in managing aggression, as well as in all breakaway domains 8 weeks after the training.

17. Meng et al., 2023 [67]	China	Cross-sectional	Emergency physicians (10,457)	Physical and verbal violence General self-efficacy	—	Items developed by the authors	Chinese version of general self-efficacy scale [68]	No theoretical framework was adopted	Self-efficacy was negatively correlated with physical and verbal violence.

18. Nawaz et al., 2021 [69]	Pakistan	Cross-sectional	Nurses in a hospital (146)	Incivility Self-efficacy Organizational citizenship behavior	Psychological capital	Workplace incivility scale [70]	Psychological capital questionnaire [42]	Conservation of resources (COR) theory [17]	Organizational citizenship behavior was negatively associated with incivility and positively associated with self-efficacy. Incivility moderated the association between psychological capital and organizational citizenship behavior.

19. Okundolor et al., 2021 [71]	USA	Nonrandomized controlled trials	Emergency psychiatric nurses (80)	Multifaceted intervention and training Physical assaults Self-efficacy in managing aggression		Items developed by the authors	Items developed by the authors	The failure mode and effects analysis (FMEA) model for improvement [72]	After at least two drills, self-efficacy increased from 75% to 95%. One year after the intervention, harmful staff assaults decreased by 75% and then dropped to 0.

20. Wu et al., 2019 [73]	Taiwan	Nonrandomized controlled trials	Emergency healthcare workers (35)	Simulation training Self-efficacy responses to workplace violence		Healthcare provider's response to workplace violence items developed by the authors	Healthcare provider's self-efficacy when responding to workplace violence scale developed by the authors	Experiential learning theory (ELT) [74]	The training significantly increased self-efficacy levels immediately after the training and at the follow-up (2 weeks later), as well as improved the ability to respond to workplace violence.

21. Yan et al., 2022 [75]	China	Cross-sectional	Emergency physicians (10,457)	Verbal aggression General self-efficacy Perceived medical error		Chinese version of effort–reward imbalance [76]	Chinese version of general self-efficacy scale [77]	No theoretical framework was adopted	Verbal violence was positively associated with medical errors, while self-efficacy was negatively associated with medical errors.

22. Yao et al., 2014 [78]	China	Cross-sectional	Physicians in a hospital (758)	Workplace violence (experienced and witnessed) General self-efficacy Occupational stress	Job satisfaction Job initiative	Workplace violence scale [79]	Chinese version of General Self-efficacy Scale [77]	Interactional theory [80]	Self-efficacy was negatively associated with workplace violence and occupational stress and positively associated with job satisfaction and initiative. Self-efficacy moderated the association between workplace violence and occupational stress, as well as workplace violence and job satisfaction, but not with job initiative

23. Yaranon et al.2024 [81]	Ireland	Design lagged	Healthcare workers in primary and residential care facilities (153)	Incivility and verbal aggression General self-efficacy Affective ill-being	Resilience	Items developed by the authors	General Self-Efficacy Scale [77]	Job-demand resources (JD-R) model [82]	Outsider mistreatment at T2 was not associated with affective ill-being at T3, and the interaction with self-efficacy at T1 was marginally significant. For those with low self-efficacy, the association between mistreatment and affective ill-being was positive (but not significant).

24. Young et al., 2022 [83]	Australia	Nonrandomized controlled trials	Mental healthcare workers (122)	Simulation training Self-efficacy in leadership, management, teamwork, and communication		Items developed by previous authors [84]	Items developed by previous authors [84]	Experiential learning theory (ELT) [74]	The intervention increased self-efficacy levels post-test, which remained steady at follow-up (3 months later). The leadership/management domain showed the greatest improvement.

**Table 2:** Quality assessment of included studies using mixed method appraisal tools (MMAT), version 2018.

	Screening questions		Quantitative randomized controlled trials					Quantitative nonrandomized studies					Quantitative descriptive studies					Comments
	S1.	S2.	2.1	2.2	2.3	2.4	2.5	3.1	3.2	3.3	3.4	3.5	4.1	4.2	4.3	4.4	4.5	
1. Ajaz et al., 2016 [36]	Y	Y						CT	N	CT	N	Y						Small sample (*n* = 9) No description of measures No control group

2. Amara et al., 2024 [37]	Y	Y						N	Y	Y	N	N						Nonprobabilistic sampling strategy No control group

3. Deng et al., 2023 [40]	Y	Y											Y	Y	Y	CT	Y	Unreported response rate

4. Flynn et al., 2018 [43]	Y	Y						CT	Y	CT	Y	CT						Unreported eligibility criteria Unreported response rate Exposure not designed by researchers

5. Gates et al., 2005 [45]	Y	Y						CT	Y	Y	CT	Y						Group allocation and sample size per condition not clearly reported Environmental confounders present

6. Gilardi et al., 2020 [46]	Y	Y											Y	CT	Y	Y	Y	Unreported eligibility criteria

7. Gordon et al., 2021 [50]	Y	Y						Y	Y	CT	N	Y						Questionnaire not validated Unreported response rate No control group

8. Goussinsky, 2020 [51]	Y	Y											Y	CT	Y	Y	Y	Unreported eligibility criteria

9. Goussinsky, 2023 [54]	Y	Y											Y	CT	Y	Y	Y	Unreported eligibility criteria

10. Goussinsky and Livne, 2019 [58]	Y	Y											Y	Y	Y	Y	Y	

11. Hatfield et al., 2022 [60]	Y	Y						N	Y	CT	CT	N						Unreported eligibility criteria and low representativeness Low response rate (37%) Mixture of same and new participants in the follow-up No control group

12. Hills, 2008 [61]	Y	Y											Y	CT	Y	Y	Y	Unreported eligibility criteria

13. Hogh et al., 2008 [62]	Y	Y											Y	CT	Y	CT	Y	Unreported eligibility criteria Unreported response rate

14. Irvine et al., 2012 [63]	Y	Y	CT	Y	Y	CT	Y											Unreported randomization process Unreported blinded assessment

15. Irvine et al., 2007 [65]	Y	Y	CT	Y	Y	CT	Y											Unreported randomization process No control of screening criteria Unreported blinded assessment

16. Lamont et al., 2012 [66]	Y	Y						CT	Y	CT	CT	Y						Unreported eligibility criteria Questionnaire not validated High attrition rate (17%) No control group

17. Meng et al., 2023 [67]	Y	Y											Y	CT	Y	Y	Y	Unreported eligibility criteria

18. Nawaz et al., 2021 [69]	Y	Y						Y	Y	Y	Y	Y						

19. Okundolor et al., 2021 [71]	Y	Y						CT	Y	CT	CT	Y						Unreported sample's sociodemographic characteristics Unreported response rate No control group

20. Wu et al., 2019 [73]	Y	Y						Y	Y	Y	CT	Y						No control group

21. Yan et al., 2022 [75]	Y	Y											Y	CT	Y	Y	Y	Unreported eligibility criteria

22. Yao et al., 2014 [78]	Y	Y											Y	CT	Y	Y	Y	Unreported eligibility criteria

23. Yaranon et al., 2024 [81]	Y	Y						Y	Y	Y	Y	Y						

24. Young et al., 2022 [83]	Y	Y						CT	Y	CT	N	Y						Unreported eligibility criteria Low response rate at follow-up (20%) No control group

*Note:* Screening questions: (S1) Are there clear research questions? (S2) Do the collected data allow to address the research questions? Quantitative randomized controlled trials: (2.1.) Is randomization appropriately performed? (2.2.) Are the groups comparable at baseline? (2.3.) Are there complete outcome data? (2.4.) Are outcome assessors blinded to the intervention provided? (2.5.) Did the participants adhere to the assigned intervention? Quantitative nonrandomized studies: (3.1.) Are the participants representative of the target population? (3.2.) Are measurements appropriate? (3.3.) Are there complete outcome data? (3.4.) Are the confounders accounted for in the design and analysis? (3.5.) During the study period, is the intervention administered (or exposure occurred) as intended? Quantitative descriptive studies: (4.1.) Is the sampling strategy relevant to address the research questions? (4.2.) Is the sample representative of the target population? (4.3) Are the measurements appropriate? (4.4.) Is the risk of nonresponse bias low? (4.5.) Is the statistical analysis appropriate to answer the research question.

Abbreviations: CT, can't tell; N, no; Y, yes.

## Data Availability

The original contributions presented in the study are included in the article. Further inquiries can be directed to the corresponding author.

## Supporting Information

The supporting information includes additional tables that support the findings of this review.

Supporting Information 1 Supporting Table 1. Prisma checklist.

Supporting Information 2 Supporting Table 2. Database-specific search strings.

Supporting Information 3 Supporting Table 3. List of excluded studies.

Supporting Information 4 Supporting Table 4. Certainty of evidence assessment and summary of findings based on GRADE.
