# Supplementary material for: Empowering Healthcare Heroes: Unveiling the Impact of Self-Efficacy on Combating Outsider Mistreatment—A Systematic Review
Source: J Nurs Manag. 2025 Sep 29;2025:7052173. doi: 10.1155/jonm/7052173 (PMC12500376; doi:10.1155/jonm/7052173)
Supplement: Supporting Information 2 — Supporting Table 2. Database-specific search strings. [file 7052173.f2.pdf]

Supplementary Table 2. Database-Specific Search Strings

DATABASE	SEARCH STRING
PsycInfo	AB ("health personnel" OR “healthcare workers” OR “health care workers” OR “healthcare professionals” OR “health care professionals” OR “healthcare providers” OR “health care providers”) AND AB (incivility OR rudeness OR "uncivil behavior" OR "uncivil behaviors" OR disrespect OR discourtesy OR violence OR aggression OR "verbal aggression" OR "physical aggression" OR mistreatment OR abuse OR harassment) AND AB (self-efficacy OR “self efficacy” OR “general self-efficacy” OR “general self efficacy” OR “work-related self-efficacy” OR “work-related self efficacy” OR “occupational self-efficacy” OR “occupational self efficacy” OR “emotional self-efficacy” OR “emotional self efficacy” OR “regulatory self-efficacy” OR “regulatory self efficacy” OR “self-efficacy beliefs” OR “self efficacy beliefs”)
PubMed	("Health Personnel"[MeSH] OR “healthcare workers”[Title/Abstract] OR “health care workers”[Title/Abstract] OR “healthcare professionals”[Title/Abstract] OR “health care professionals”[Title/Abstract] OR “healthcare providers”[Title/Abstract] OR “health care providers”[Title/Abstract]) AND (incivility[Title/Abstract] OR rudeness[Title/Abstract] OR "uncivil behavior*"[Title/Abstract] OR disrespect[Title/Abstract] OR discourtesy[Title/Abstract] OR violence[Title/Abstract] OR aggression[Title/Abstract] OR "verbal aggression"[Title/Abstract] OR "physical aggression"[Title/Abstract] OR mistreatment[Title/Abstract] OR abuse[Title/Abstract] OR harassment[Title/Abstract]) AND (“Self Efficacy”[MeSH] OR self-efficacy[Title/Abstract] OR “general self-efficacy”[Title/Abstract] OR “general self efficacy”[Title/Abstract] OR “work-related self-efficacy”[Title/Abstract] OR “work-related self-efficacy”[Title/Abstract] OR “occupational self-efficacy”[Title/Abstract] OR “occupational self efficacy”[Title/Abstract] OR “emotional self-efficacy”[Title/Abstract] OR “emotional self efficacy”[Title/Abstract] OR “regulatory self-efficacy”[Title/Abstract] OR “regulatory self efficacy”[Title/Abstract] OR “self-efficacy beliefs”[Title/Abstract] OR “self efficacy beliefs”[Title/Abstract])
Scopus	(TITLE-ABS-KEY("health personnel") OR TITLE-ABS-KEY("healthcare workers") OR TITLE-ABS-KEY("health care workers") OR TITLE-ABS-KEY("healthcare professionals") OR TITLE-ABS-KEY("health care professionals") OR TITLE-ABS-KEY("healthcare providers") OR TITLE-ABS-KEY("health care providers")) AND (TITLE-ABS-KEY(incivility) OR TITLE-ABS-KEY(rudeness) OR TITLE-ABS-KEY("uncivil behaviors") OR TITLE-ABS-KEY("uncivil behavior") OR TITLE-ABS-KEY(disrespect) OR TITLE-ABS-KEY(discourtesy) OR TITLE-ABS-KEY(violence) OR TITLE-ABS-KEY(aggression) OR TITLE-ABS-KEY("verbal aggression") OR TITLE-ABS-KEY("physical aggression") OR TITLE-ABS-KEY(mistreatment) OR TITLE-ABS-KEY(abuse) OR TITLE-ABS-KEY(harassment)) AND (TITLE-ABS-KEY(self-efficacy) OR TITLE-ABS-KEY("self efficacy") OR TITLE-ABS-KEY("general self-efficacy") OR TITLE-ABS-KEY("general self efficacy") OR TITLE-ABS-KEY("work-related self-efficacy") OR TITLE-ABS-KEY("work-related self efficacy") OR TITLE-ABS-KEY("occupational self-efficacy") OR TITLE-ABS-KEY("occupational self efficacy") OR TITLE-ABS-KEY("emotional self-efficacy") OR TITLE-ABS-KEY("emotional self efficacy") OR TITLE-ABS-KEY("regulatory self-efficacy") OR TITLE-ABS-KEY("regulatory self efficacy") OR TITLE-ABS-KEY("self-efficacy beliefs") OR TITLE-ABS-KEY("self efficacy beliefs"))
