# Supplementary material for: Empowering Healthcare Heroes: Unveiling the Impact of Self-Efficacy on Combating Outsider Mistreatment—A Systematic Review
Source: J Nurs Manag. 2025 Sep 29;2025:7052173. doi: 10.1155/jonm/7052173 (PMC12500376; doi:10.1155/jonm/7052173)
Supplement: Supporting Information 3 — Supporting Table 3. List of excluded studies. [file 7052173.f3.pdf]

Supplementary Table 3. List of exlcuded studies

Author(s), year	Country	Study design	Subjects/setting (n)	Observed variables	Reason for exclusion	References
**1. Cramer et al., 2020**	United Kingdom	Cross-sectional	Healthcare workers in psychiatric wards (170)	Coping self-efficacy<br>Mental health<br>Occupational health	No assessment of outsider mistreatment	Cramer RJ, Ireland JL, Hartley V, Long MM, Ireland CA, Wilkins T. Coping, mental health, and subjective well-being among mental health staff working in secure forensic psychiatric settings: Results from a workplace health assessment. Psychological Services 2019;17:160–9. https://doi.org/10.1037/ser0000354
**2. Daly & Coffy, 2010**	Ireland	Cross-sectional	Nurses and care assistants in long-term care settings (114)	Elder abuse	No assessment of outsider mistreatment and self-efficacy	Daly J, Coffey A. Staff perceptions of elder abuse. Nursing Older People 2010;22:33–7. https://doi.org/10.7748/nop2010.05.22.4.33.c7735
**3. Fida et al., 2018**	Canada	Design Lagged	Nurses (596)	Workplace incivility<br>Occupational Self-efficacy<br>Burnout symptoms<br>Turnover intention	Source of incivility: Coworkers and supervisors	Fida R, Laschinger HKS, Leiter MP. The protective role of self-efficacy against workplace incivility and burnout in nursing. Health Care Management Review 2018;43:21–9. https://doi.org/10.1097/hmr.0000000000000126
**4. Gerhart et al., 2017**	USA	Non randomized controlled trials	Healthcare workers in oncology wards (12)	Training program<br>Patients' anger<br>Self-efficacy in managing patient anger	The intervention did not focus on managing outsider mistreatment	Gerhart JI, Varela VS, Burns JW. Brief training on patient anger Increases oncology providers' Self-Efficacy in communicating with angry patients. Journal of Pain and Symptom Management 2017;54:355-360.e2. https://doi.org/10.1016/j.jpainsymman.2017.07.039
**5. Heckemann et al., 2019**	Switzerland, Austria and Germany	Cross-sectional	Nurses in general and psychiatric hospitals (446)	Team efficacy in managing aggression<br>Organisational safety culture	No assessment of outsider mistreatment	Heckemann B, Hahn S, Halfens RJG, Richter D, Schols JMGA. Patient and visitor aggression in healthcare: A survey exploring organisational safety culture and team efficacy. Journal of Nursing Management 2019;27:1039–46. https://doi.org/10.1111/jonm.12772
**6. Hensel et al., 2014**	Canada	Cross-sectional	Healthcare workers in residential and hospital facilities (356)	Aggression<br>Self-efficacy in managing aggression<br>Burnout symptoms	No association tested between self-efficacy and mistreatment, nor with individual or occupational well-being	Hensel JM, Lunsky Y, Dewa CS. The mediating effect of severity of client aggression on burnout between hospital inpatient and community residential staff who support adults with intellectual disabilities. Journal of Clinical Nursing 2014;23:1332–41. https://doi.org/10.1111/jocn.12387

**7. Huang et al., 2023**	China	Cross-sectional	Emergency nurses (825)	General self-efficacy<br>Organizational support<br>Resilience<br>Cognitive reappraisal	Outsider mistreatment used only as exclusion criterion; not analysed with other variables	Huang H, Su Y, Liao L, Li R, Wang L. Perceived organizational support, self-efficacy and cognitive reappraisal on resilience in emergency nurses who sustained workplace violence: A mediation analysis. Journal of Advanced Nursing 2023;80:2379–91. https://doi.org/10.1111/jan.16006
**8. Lasater et al., 2015**	USA	Non randomized controlled trials	Nurses (94)	Educational Intervention<br>Incivility<br>Self-efficacy	Source of incivility: co-workers	Lasater K, Mood L, Buchwach D, Dieckmann NF. Reducing Incivility in the Workplace: Results of a Three-Part Educational Intervention. The Journal of Continuing Education in Nursing 2014;46:15–24. https://doi.org/10.3928/00220124-20141224-01
**9. Martin & Daffern, 2006**	Australia	Cross-sectional	Clinicians in a forensic psychiatric hospital (125)	Confidence in coping with aggression<br>Clinician perceptions of personal safety	No assessment of outsider mistreatment	Martin T, Daffern M. Clinician perceptions of personal safety and confidence to manage inpatient aggression in a forensic psychiatric setting. Journal of Psychiatric and Mental Health Nursing 2006;13:90–9. https://doi.org/10.1111/j.1365-2850.2006.00920.x
**10. McConville & Lane, 2006**	United Kingdom	Non randomized controlled trials	Nursing students (145)	On-line educational training<br>Self-efficacy in managing aggression	The intervention addressed general difficult situations without specifically focusing on outsider mistreatment	McConville SA, Lane AM. Using on-line video clips to enhance self-efficacy toward dealing with difficult situations among nursing students. Nurse Education Today 2006;26:200–8. https://doi.org/10.1016/j.nedt.2005.09.024
**11. McLaughlin et al., 2010**	United Kingdom	Non randomized controlled trials	Healthcare workers in a psychiatric ward (18)	Training program<br>Verbal aggression	The effectiveness of the training was assessed using qualitative methods	McLaughlin S, Bonner G, Mboche C, Fairlie T. A pilot study to test an intervention for dealing with verbal aggression. British Journal of Nursing 2010;19:489–94. https://doi.org/10.12968/bjon.2010.19.8.47638
**12. McNiel et al., 2008**	USA	Non randomized controlled trials	Psychiatry and psychology trainees (45)	Training program in managing risk of violence and suicide<br>Competences in risk assessment	The training focused on violence risk assessment without directly addressing the management of outsider aggression	McNiel DE, Chamberlain JR, Weaver CM, Hall SE, Fordwood SR, Binder RL. Impact of clinical training on violence risk assessment. American Journal of Psychiatry 2008;165:195–200. https://doi.org/10.1176/appi.ajp.2007.06081396

**13. Molero Jurado et al., 2019**	Spain	Cross-sectional	Nurses (1777)	General self-efficacy<br>Emotional Intelligence<br>Perceived stress	No assessment of outsider mistreatment	Del Mar Molero Jurado M, Del Carmen Pérez-Fuentes M, Ruiz NFO, Del Mar Simón Márquez M, Linares JJG. Self-Efficacy and emotional intelligence as predictors of perceived stress in nursing professionals. Medicina 2019;55:237. https://doi.org/10.3390/medicina55060237
**14. Needham et al., 2005**	Switzerland	Randomized controlled trials	Nurses in psychiatric wards (114)	Training in aggression management<br>Nurses' perceptions and attitudes towards aggression	No assessment of self-efficacy	Needham I, Abderhalden C, Halfens RJG, Dassen T, Haug HJ, Fischer JE. The effect of a training course in aggression management on mental health nurses' perceptions of aggression: a cluster randomised controlled trial. International Journal of Nursing Studies 2005;42:649–55. https://doi.org/10.1016/j.ijnurstu.2004.10.003
**15. Oh et al., 2016**	South Korea	Cross-sectional	Nurses (442)	Workplace bullying<br>Lateral violence<br>Job stress<br>Intention to leave<br>Job safety	Sources of mistreatment: supervisor and coworkers.<br>No assessment of self-efficacy	Oh H, Uhm DC, Yoon YJ. Workplace bullying, job stress, intent to leave, and nurses' perceptions of patient safety in South Korean hospitals. Nursing Research 2016;65:380–8. https://doi.org/10.1097/nnr.0000000000000175
**16. Palumbo, 2018**	USA	Non randomized controlled trials	Nursing students (110)	Educational training<br>Incivility<br>Self-efficacy in managing incivility	Source of incivility: co-workers	Palumbo R. Incivility in nursing education: An intervention. Nurse Education Today 2018;66:143–8. https://doi.org/10.1016/j.nedt.2018.03.024
**17. Pariona Cabrera et al., 2023**	Australia	Cross-sectional	Nurses and personal care assistants (254)	Physical violence<br>Psychological Capital<br>Mental health	Self-efficacy not isolated in analysis	Pariona-Cabrera P, Meacham H, Tham TL, Cavanagh J, Halvorsen B, Holland P, et al. The buffering effects of psychological capital on the relationship between physical violence and mental health issues of nurses and personal care assistants working in aged care facilities. Health Care Management Review 2023;48:42–51. https://doi.org/10.1097/hmr.0000000000000348
**18. Pines et al., 2021**	USA	Non randomized controlled trials	Healthcare workers (43)	Training in communication<br>Experience of workplace violence<br>Self-efficacy in communication to aggressive patients	Mixed-methods; qualitative data analysed.	Pines R, Giles H, Watson B. Managing patient aggression in healthcare: Initial testing of a communication accommodation theory intervention. Psychology of Language and Communication 2021;25:62–81. https://doi.org/10.2478/plc-2021-0004

				Patient cooperation<br>Communication accommodation strategies		
**19. Schimd et al., 2020**	Switzerland	Randomized controlled trials	Professional caregivers (47)	Trauma-informed care intervention<br>Cortisol levels<br>Physical aggression	Sample: not healthcare workers<br>Self-efficacy was not evaluated<br>No assessment of self-efficacy	Schmid M, Lüdtke J, Dolitzsch C, Fischer S, Eckert A, Fegert JM. Effect of trauma-informed care on hair cortisol concentration in youth welfare staff and client physical aggression towards staff: results of a longitudinal study. BMC Public Health 2020;20. https://doi.org/10.1186/s12889-019-8077-2
**20. Shimosato & Kinoshita, 2018**	Japan	Cross-sectional	Nurses in psychiatric ward (313)	Self-efficacy in managing aggression<br>Nurses' levels of anger<br>Nurses' aggressiveness<br>Nurses' attitudes toward aggression<br>Self-efficacy in managing aggression	No assessment of outsider mistreatment	Shimosato S, Kinoshita A. Degree of Anger during Anger-Generating Situations among Psychiatric Staff Nurses: Association between nurses' attitudes toward service users' aggression and confidence in intervening in aggressive situations. Journal of Psychosocial Nursing and Mental Health Services 2018;56:51–9. https://doi.org/10.3928/02793695-20180322-02
**21. Verhaeghe et al., 2014**	Belgium	Cross-sectional	Nurses in psychiatric wards (219)	Self-efficacy in managing aggression<br>Attitudes toward aggression<br>Burnout symptoms<br>Secondary traumatic stress<br>Compassion satisfaction	No assessment of outsider mistreatment	Verhaeghe S, Duprez V, Beeckman D, Leys J, Van Meijel B, Van Hecke A. Mental Health Nurses' Attitudes and Perceived Self-Efficacy toward Inpatient Aggression: A Cross-Sectional Study of Associations with Nurse-Related Characteristics. Perspectives in Psychiatric Care 2014;52:12–24. https://doi.org/10.1111/ppc.12097
**22. Vieira-Meyer et al., 2023**	Brazil	Cross-sectional	Community health care workers (1935)	Violence<br>General self-efficacy<br>COVID-19-related anxiety<br>Mental health<br>Quality of life<br>Perceived social support	Not in English	Vieira-Meyer APGF, Farias SF, Forte FDS, Costa MS, Guimarães JMX, Morais APP, et al. Saúde mental de agentes comunitários de saúde no contexto da COVID-19. Ciência & Saúde Coletiva 2023;28:2363–76. https://doi.org/10.1590/1413-81232023288.06462023
**23. Yada et al., 2020**	Japan	Cross-sectional	Nurses and assistant nurses (132)	General self-efficacy	Outsider mistreatment was not evaluated	Yada H, Abe H, Odachi R, Adachi K. Exploration of the factors related to self-efficacy among psychiatric nurses. PLoS ONE 2020;15:e0230740. https://doi.org/10.1371/journal.pone.0230740
