# Supplementary material for: Empowering Healthcare Heroes: Unveiling the Impact of Self-Efficacy on Combating Outsider Mistreatment—A Systematic Review
Source: J Nurs Manag. 2025 Sep 29;2025:7052173. doi: 10.1155/jonm/7052173 (PMC12500376; doi:10.1155/jonm/7052173)
Supplement: Supporting Information 4 — Supporting Table 4. Certainty of evidence assessment and summary of findings based on GRADE. [file 7052173.f4.pdf]

Supplementary Table 4. Certainty of Evidence Assessment and Summary of Findings Based on GRADE

Certainty assessment							Summary of findings			
N° of studies	Study design	Risk of bias	Inconsistency	Indirectness	Imprecision	Publication bias	N° of participants	Impact	Certainty	Importance
**Outcome: relationship between self-efficacy and outsider mistreatment**										
11	Non-randomized studies	Not serious	Not serious	Not serious	Serious[a]	Undetected	18553	General self-efficacy was consistently associated with lower levels of outsider mistreatment across studies. In contrast, domain-specific self-efficacy showed weaker and more inconsistent associations, likely due to variability in assessment tools and specific domains evaluated. Methodological heterogeneity and contextual factors (e.g., care settings, healthcare worker and patient characteristics) further contributed to mixed results.	⨁◯◯◯ Very low[a]	CRITICAL
**Outcome: effect of self-efficacy on the relationship between outsider mistreatment and occupational well-being**										
10	Non-randomized studies	Not serious	Not serious	Not serious	Serious[a]	Undetected	18213	Higher self-efficacy is associated with reduced occupational stress and burnout, and more favourable job-related outcomes (e.g., job satisfaction, fewer errors). However, its protective role varies depending on contextual resources. It may be attenuated in resource-constrained or high-stress contexts (e.g., under conditions of prolonged outsider mistreatment).	⨁◯◯◯ Very low[a]	CRITICAL
**Outcome: effectiveness of aggression management interventions in increasing self-efficacy**										
11	Non-randomized studies (9);	Serious[b]	Not serious	Not serious	Serious[c]	Undetected	978	Targeted interventions (e.g., simulation-based, educational, and physical training) demonstrated	⨁◯◯◯ Very low[b,c]	CRITICAL

Randomized control studies (2)	improvements in healthcare workers' self-efficacy in managing outsider mistreatment. Organizationally embedded approaches showed more sustained effects. Nonetheless, confidence is limited due to lack of validated tools and absence of long-term follow-up in several studies.

Explanations

a. Imprecision: variability in measurement instruments and differences in contextual factors (e.g., healthcare settings, participant characteristics) reduced the consistency and reliability of effect estimates, contributing to imprecision in the findings.

b. Risk of bias: ss evaluated using the Mixed Method Appraisal Tools (MMAT; see Table 2), several studies did not fully meet the methodological criteria for good or high quality, leading to concerns regarding internal validity due to reliance on self-reported outcomes, and use of non-validated or ad hoc measures.

c. Imprecision: small sample sizes, absence of long-term follow-up, and reliance on non-validated or ad hoc measures, which increased the uncertainty around the estimated effects, warranting a downgrade for imprecision.
